# Supplementary material for: An Approach for the Identification of Targets Specific to Bone Metastasis Using Cancer Genes Interactome and Gene Ontology Analysis
Source: PLoS One. 2012 Nov 14;7(11):e49401. doi: 10.1371/journal.pone.0049401 (PMC3498148; doi:10.1371/journal.pone.0049401)
Supplement: Table S1 — Hub genes (Top25–Top200) of Cancer Genes Network. (PDF) [file pone.0049401.s001.pdf]

Table S1. Hub genes (Top25—Top200) of Cancer Genes Network.

Top25 hubs (31)	Top50 hubs (60)	Top75 hubs (92)	Top100 hubs (125)	Top125 hubs (158)	Top150 hubs (185)	Top175 hubs (213)	Top200 hubs (247)
ABL1	ABL1	ABL1	ABL1	ABL1	ABL1	ABL1	ABL1
AKT1	AKT1	ACTB	ACTB	ACTB	ACTB	ACTB	ACTB
AR	AR	AKT1	AKT1	AKT1	AKT1	ACVR1	ACVR1
BRCA1	BCL2	APP	APP	APP	APP	AKT1	AKT1
CASP3	BRCA1	AR	AR	AR	AR	APP	APP
CREBBP	CASP3	BCL2	BCL2	AXIN1	ATM	AR	AR
CSNK2A1	CAV1	BRCA1	BRCA1	BCL2	AXIN1	ATM	ATF2
CTNNB1	CBL	CASP3	CASP3	BRCA1	BAT3	AXIN1	ATM
EGFR	CDK2	CASP8	CASP8	BTK	BCL2	BAT3	AXIN1
EP300	CREBBP	CAV1	CAV1	CALR	BCR	BCAR1	BAD
ESR1	CSNK2A1	CBL	CBL	CASP3	BRCA1	BCL2	BAT3
FYN	CTNNB1	CDK2	CDK2	CASP8	BTK	BCR	BCAR1
GRB2	DLG4	CHUK	CDKN1A	CAV1	CADM1	BIRC2	BCL2
HDAC1	EGFR	CREBBP	CDKN1B	CBL	CALR	BRCA1	BCR
JUN	EP300	CRK	CEBPB	CCNB1	CASP3	BRCA2	BIRC2
MAPK1	ESR1	CSNK2A1	CHUK	CCND1	CASP8	BTK	BMPR1B
MAPK3	FLNA	CTNNB1	CREBBP	CDC42	CAV1	CADM1	BRCA1
PRKACA	FYN	DLG4	CRK	CDK2	CBL	CALR	BRCA2
PRKCA	GRB2	EEF1A1	CSNK2A1	CDK9	CCNB1	CASP3	BTK
RAF1	GSK3B	EGFR	CSNK2A2	CDKN1A	CCND1	CASP8	BTRC
RB1	HDAC1	EP300	CTNNB1	CDKN1B	CDC42	CAV1	CADM1
RELA	HSP90AA1	ESR1	DLG4	CEBPB	CDK2	CBL	CALR
SMAD2	INSR	EWSR1	DYNLL1	CHUK	CDK4	CCNB1	CASP3
SMAD3	JAK2	FLNA	EEF1A1	CREBBP	CDK9	CCND1	CASP8
SMAD4	JUN	FYN	EGFR	CRK	CDKN1A	CDC42	CAV1
SRC	LCK	GNAI2	EP300	CRKL	CDKN1B	CDK2	CBL
STAT3	LYN	GRB2	EPHB2	CSNK2A1	CEBPB	CDK4	CCNB1
TGFBR1	MAPK1	GSK3B	ERBB2	CSNK2A2	CHUK	CDK9	CCND1
TP53	MAPK14	HDAC1	ESR1	CTNNB1	CREB1	CDKN1A	CD44
YWHAG	MAPK3	HRAS	EWSR1	DAXX	CREBBP	CDKN1B	CDC42
YWHAZ	NFKB1	HSP90AA1	FLNA	DLG4	CRK	CEBPB	CDH1
	NR3C1	IGF1R	FOS	DYNLL1	CRKL	CHUK	CDK2
	PAK1	INSR	FYN	EEF1A1	CSK	CREB1	CDK4
	PCNA	IRS1	GNAI2	EGFR	CSNK2A1	CREBBP	CDK9
	PLCG1	JAK1	GRB2	EP300	CSNK2A2	CRK	CDKN1A
	PRKACA	JAK2	GSK3B	EPB41L3	CTNNB1	CRKL	CDKN1B
	PRKCA	JUN	HDAC1	EPHB2	DAXX	CSK	CDKN2A
	PRKCD	KAT5	HDAC2	ERBB2	DLG4	CSNK2A1	CEBPB
	PTK2	KIT	HIF1A	ESR1	DYNLL1	CSNK2A2	CHD3

	PTPN11	LCK	HMGB1	EWSR1	E2F1	CTNNB1	CHUK
	RAF1	LRP1	HRAS	FLNA	EEF1A1	DAXX	CREB1
	RB1	LYN	HSP90AA1	FN1	EGFR	DLG4	CREBBP
	RELA	MAPK1	HTT	FOS	EP300	DYNLL1	CRK
	SMAD2	MAPK14	IGF1R	FYN	EPB41L3	E2F1	CRKL
	SMAD3	MAPK3	IKBKB	GNAI2	EPHB2	EEF1A1	CSK
	SMAD4	MAPK8	IKBKG	GNB2L1	ERBB2	EGFR	CSNK2A1
	SP1	MDM2	INSR	GRB2	ESR1	EP300	CSNK2A2
	SRC	MYC	IRS1	GSK3B	ESR2	EPB41L3	CSNK2B
	STAT1	NCK1	JAK1	HCK	EWSR1	EPHB2	CTNNB1
	STAT3	NFKB1	JAK2	HDAC1	FLNA	EPOR	DAXX
	TGFBR1	NFKBIA	JUN	HDAC2	FN1	ERBB2	DLG4
	TP53	NOTCH1	KAT5	HDAC3	FOS	ESR1	DOK1
	TRAF2	NR3C1	KIT	HIF1A	FYN	ESR2	DVL1
	TRAF6	PAK1	LCK	HIPK2	GNAI2	ETS1	DYNLL1
	UBB	PCNA	LRP1	HMGB1	GNB2L1	EWSR1	E2F1
	UBE2I	PLCG1	LYN	HRAS	GRB2	FGFR1	EEF1A1
	VIM	PML	MAPK1	HSP90AA1	GSK3B	FLNA	EGFR
	YWHAB	PRKACA	MAPK14	HSPA1A	HCK	FN1	EP300
	YWHAG	PRKCA	MAPK3	HTT	HDAC1	FOS	EPB41
	YWHAZ	PRKCD	MAPK8	IGF1R	HDAC2	FYN	EPB41L3
		PRKCZ	MDM2	IKBKB	HDAC3	GNA13	EPHB2
		PTK2	MYC	IKBKG	HIF1A	GNAI2	EPOR
		PTK2B	NCK1	INSR	HIPK2	GNB2L1	ERBB2
		PTPN11	NCOA1	IRS1	HMGB1	GRB2	ERBB4
		PTPN6	NCOR2	JAK1	HRAS	GSK3B	ESR1
		RAC1	NFKB1	JAK2	HSP90AA1	HCK	ESR2
		RAF1	NFKBIA	JUN	HSPA1A	HDAC1	ETS1
		RASA1	NFKBIB	KAT2B	HTT	HDAC2	EWSR1
		RB1	NOTCH1	KAT5	IGF1R	HDAC3	FADD
		RELA	NR3C1	KIT	IKBKB	HIF1A	FGFR1
		SMAD1	PAK1	LCK	IKBKG	HIPK2	FLNA
		SMAD2	PCNA	LRP1	INSR	HMGB1	FN1
		SMAD3	PDGFRB	LYN	IRS1	HRAS	FOS
		SMAD4	PIN1	MAP3K7	ITGB1	HSP90AA1	FYN
		SP1	PLCG1	MAPK1	JAK1	HSPA1A	GNA13
		SRC	PML	MAPK14	JAK2	HTT	GNAI2
		STAT1	POLR2A	MAPK3	JUN	IGF1R	GNB2L1
		STAT3	PRKACA	MAPK8	KAT2B	IGF2	GRB2
		SUMO4	PRKCA	MBP	KAT5	IKBKB	GSK3B
		SYK	PRKCD	MDM2	KDR	IKBKG	HCK
		TGFBR1	PRKCZ	MET	KIT	INSR	HDAC1
		TP53	PTCH1	MYC	LCK	IRS1	HDAC2

		TRAF2	PTEN	MYOD1	LRP1	ITGB1	HDAC3
		TRAF6	PTK2	NCK1	LYN	ITGB4	HGS
		UBB	PTK2B	NCOA1	MAP3K14	JAK1	HIF1A
		UBE2I	PTPN11	NCOA3	MAP3K7	JAK2	HIPK2
		VAV1	PTPN6	NCOR1	MAPK1	JUN	HMGB1
		VIM	RAC1	NCOR2	MAPK14	KAT2B	HRAS
		YWHAB	RAD51	NFKB1	MAPK3	KAT5	HSP90AA1
		YWHAG	RAF1	NFKBIA	MAPK8	KDR	HSPA1A
		YWHAZ	RASA1	NFKBIB	MAPT	KHDRBS1	HTT
		ZBTB16	RB1	NOTCH1	MBP	KIT	IGF1R
			RELA	NR3C1	MDM2	KPNB1	IGF2
			RXRA	PAK1	MET	KRT18	IKBKB
			SIN3A	PCNA	MYC	LCK	IKBKG
			SMAD1	PDGFRB	MYOD1	LRP1	IL6ST
			SMAD2	PIAS1	NCK1	LRP6	INSR
			SMAD3	PIN1	NCOA1	LYN	IRAK1
			SMAD4	PLCG1	NCOA3	MAP3K14	IRS1
			SMURF2	PML	NCOR1	MAP3K5	IRS2
			SP1	POLR2A	NCOR2	MAP3K7	ITGB1
			SRC	PPP2CA	NDRG1	MAPK1	ITGB3
			STAT1	PRKACA	NFKB1	MAPK14	ITGB4
			STAT3	PRKAR2A	NFKBIA	MAPK3	JAK1
			STAT5A	PRKCA	NFKBIB	MAPK8	JAK2
			SUMO1	PRKCD	NOTCH1	MAPK9	JUN
			SUMO4	PRKCZ	NR3C1	MAPT	KAT2B
			SVIL	PTCH1	PAK1	MBP	KAT5
			SYK	PTEN	PCNA	MDM2	KDR
			TBP	PTK2	PDGFRB	MET	KHDRBS1
			TGFBR1	PTK2B	PIAS1	MMP2	KIT
			TNFRSF1A	PTPN11	PIN1	MYC	KPNB1
			TP53	PTPN6	PLCG1	MYOD1	KRT18
			TRAF2	PTPRC	PLSCR1	NCK1	LCK
			TRAF6	RAC1	PML	NCOA1	LRP1
			UBB	RAD51	POLR2A	NCOA2	LRP6
			UBE2I	RAF1	PPP2CA	NCOA3	LYN
			VAV1	RASA1	PRKAA1	NCOR1	MAP2K1
			VIM	RB1	PRKACA	NCOR2	MAP3K14
			XRCC6	RELA	PRKAR2A	NDRG1	MAP3K5
			YWHAB	RET	PRKCA	NFKB1	MAP3K7
			YWHAG	RHOA	PRKCD	NFKBIA	MAPK1
			YWHAH	RXRA	PRKCZ	NFKBIB	MAPK14
			YWHAZ	SIN3A	PRKDC	NOTCH1	MAPK3
			ZBTB16	SMAD1	PSEN1	NR3C1	MAPK8

				SMAD2	PTCH1	PAK1	MAPK9
				SMAD3	PTEN	PCNA	MAPT
				SMAD4	PTK2	PDGFRB	MBP
				SMURF2	PTK2B	PDPK1	MDM2
				SP1	PTPN1	PIAS1	MET
				SRC	PTPN11	PIN1	MITF
				STAT1	PTPN6	PLCG1	MLLT4
				STAT3	PTPRC	PLSCR1	MMP2
				STAT5A	RAC1	PML	MMP9
				STAT5B	RAD51	POLR2A	MPP3
				SUMO1	RAF1	PPP2CA	MYC
				SUMO4	RARA	PPP2R1B	MYOD1
				SVIL	RASA1	PRKAA1	NCK1
				SYK	RB1	PRKACA	NCOA1
				TBP	RELA	PRKAR2A	NCOA2
				TGFBR1	RET	PRKCA	NCOA3
				TNFRSF1A	RHOA	PRKCD	NCOA6
				TP53	RXRA	PRKCZ	NCOR1
				TRAF2	SIN3A	PRKDC	NCOR2
				TRAF6	SKP1	PSEN1	NDRG1
				TUBB	SMAD1	PTCH1	NFKB1
				UBB	SMAD2	PTEN	NFKBIA
				UBE2I	SMAD3	PTK2	NFKBIB
				VAV1	SMAD4	PTK2B	NGFR
				VIM	SMURF2	PTPN1	NOTCH1
				XRCC6	SOCS1	PTPN11	NR3C1
				YWHAB	SOS1	PTPN6	PAK1
				YWHAE	SP1	PTPRC	PARP1
				YWHAG	SRC	RAC1	PCNA
				YWHAH	STAT1	RAD51	PDGFRB
				YWHAQ	STAT3	RAF1	PDPK1
				YWHAZ	STAT5A	RARA	PIAS1
				ZBTB16	STAT5B	RASA1	PIK3R2
					SUMO1	RB1	PIN1
					SUMO4	RELA	PLCG1
					SVIL	RET	PLSCR1
					SYK	RHOA	PML
					TBP	RUNX1	POLR2A
					TGFBR1	RXRA	PPP2CA
					TNFRSF1A	SIN3A	PPP2R1B
					TP53	SKIL	PRKAA1
					TP73	SKP1	PRKACA
					TRAF2	SMAD1	PRKAR2A

					TRAF6	SMAD2	PRKCA
					TSC2	SMAD3	PRKCD
					TUBB	SMAD4	PRKCZ
					UBB	SMAD7	PRKDC
					UBE2I	SMARCA4	PSEN1
					VAV1	SMURF1	PTCH1
					VIM	SMURF2	PTEN
					XPO1	SNCA	PTK2
					XRCC6	SOCS1	PTK2B
					YWHAB	SOS1	PTPN1
					YWHAE	SP1	PTPN11
					YWHAG	SRC	PTPN6
					YWHAH	STAT1	PTPRC
					YWHAQ	STAT3	RAC1
					YWHAZ	STAT5A	RAD51
					ZAP70	STAT5B	RAF1
					ZBTB16	SUMO1	RARA
						SUMO4	RASA1
						SVIL	RB1
						SYK	RBL1
						TBP	RELA
						TCF3	RELB
						TGFBR1	RET
						TNFRSF1A	RHOA
						TP53	RIPK1
						TP73	RUNX1
						TRAF2	RXRA
						TRAF6	SIN3A
						TSC2	SKIL
						TUBB	SKP1
						UBB	SKP2
						UBE2I	SMAD1
						VAV1	SMAD2
						VHL	SMAD3
						VIM	SMAD4
						XPO1	SMAD7
						XRCC6	SMARCA4
						YWHAB	SMURF1
						YWHAE	SMURF2
						YWHAG	SNCA
						YWHAH	SOCS1
						YWHAQ	SOS1

							YWHAZ<br>ZAP70<br>ZBTB16	SP1<br>SRC<br>STAT1<br>STAT3<br>STAT5A<br>STAT5B<br>SUMO1<br>SUMO4<br>SVIL<br>SYK<br>TBP<br>TCF3<br>TERT<br>TGFBR1<br>TGFBR2<br>TNFRSF1A<br>TP53<br>TP73<br>TRAF2<br>TRAF6<br>TSC2<br>TUBB<br>UBB<br>UBE2I<br>VAV1<br>VHL<br>VIM<br>XPO1<br>XRCC6<br>YWHAB<br>YWHAE<br>YWHAG<br>YWHAH<br>YWHAQ<br>YWHAZ<br>ZAP70<br>ZBTB16
