# Supplementary material for: An Approach for the Identification of Targets Specific to Bone Metastasis Using Cancer Genes Interactome and Gene Ontology Analysis
Source: PLoS One. 2012 Nov 14;7(11):e49401. doi: 10.1371/journal.pone.0049401 (PMC3498148; doi:10.1371/journal.pone.0049401)
Supplement: Table S2 — Details of KEGG-PIC genes (328) from KEGG PATHWAY Database. (PDF) [file pone.0049401.s002.pdf]

Table S2. Details of KEGG-PIC genes (328) from KEGG PATHWAY Database.

HGNC Gene ID	Gene Symbol	Full Name of the Gene
1630	DCC	deleted in colorectal carcinoma
836	CASP3	caspase 3, apoptosis-related cysteine peptidase (EC:3.4.22.56)
842	CASP9	caspase 9, apoptosis-related cysteine peptidase (EC:3.4.22.62)
26060	APPL1	adaptor protein, phosphotyrosine interaction, PH domain and leucine zipper containing 1
999	CDH1	cadherin 1, type 1, E-cadherin (epithelial)
1499	CTNNB1	catenin (cadherin-associated protein), beta 1, 88kDa
1496	CTNNA2	catenin (cadherin-associated protein), alpha 2
1495	CTNNA1	catenin (cadherin-associated protein), alpha 1, 102kDa
29119	CTNNA3	catenin (cadherin-associated protein), alpha 3
8312	AXIN1	axin 1
8313	AXIN2	axin 2
10297	APC2	adenomatosis polyposis coli 2
324	APC	adenomatous polyposis coli
2932	GSK3B	glycogen synthase kinase 3 beta (EC:2.7.11.1 2.7.11.26)
6932	TCF7	transcription factor 7 (T-cell specific, HMG-box)
83439	TCF7L1	transcription factor 7-like 1 (T-cell specific, HMG-box)
6934	TCF7L2	transcription factor 7-like 2 (T-cell specific, HMG-box)
51176	LEF1	lymphoid enhancer-binding factor 1
332	BIRC5	baculoviral IAP repeat containing 5
4609	MYC	v-myc myelocytomatosis viral oncogene homolog (avian)
595	CCND1	cyclin D1
7471	WNT1	wingless-type MMTV integration site family, member 1
7482	WNT2B	wingless-type MMTV integration site family, member 2B
7472	WNT2	wingless-type MMTV integration site family member 2
7473	WNT3	wingless-type MMTV integration site family, member 3
89780	WNT3A	wingless-type MMTV integration site family, member 3A
54361	WNT4	wingless-type MMTV integration site family, member 4
7474	WNT5A	wingless-type MMTV integration site family, member 5A
81029	WNT5B	wingless-type MMTV integration site family, member 5B
7475	WNT6	wingless-type MMTV integration site family, member 6
7477	WNT7B	wingless-type MMTV integration site family, member 7B
7476	WNT7A	wingless-type MMTV integration site family, member 7A
7479	WNT8B	wingless-type MMTV integration site family, member 8B
7478	WNT8A	wingless-type MMTV integration site family, member 8A
7483	WNT9A	wingless-type MMTV integration site family, member 9A

7484	WNT9B	wingless-type MMTV integration site family, member 9B
7480	WNT10B	wingless-type MMTV integration site family, member 10B
80326	WNT10A	wingless-type MMTV integration site family, member 10A
7481	WNT11	wingless-type MMTV integration site family, member 11
51384	WNT16	wingless-type MMTV integration site family, member 16
8321	FZD1	frizzled family receptor 1
8324	FZD7	frizzled family receptor 7
2535	FZD2	frizzled family receptor 2
7976	FZD3	frizzled family receptor 3
8322	FZD4	frizzled family receptor 4
8325	FZD8	frizzled family receptor 8
7855	FZD5	frizzled family receptor 5
8323	FZD6	frizzled family receptor 6
8326	FZD9	frizzled family receptor 9
11211	FZD10	frizzled family receptor 10
1856	DVL2	dishevelled, dsh homolog 2 (Drosophila)
1857	DVL3	dishevelled, dsh homolog 3 (Drosophila)
1855	DVL1	dishevelled, dsh homolog 1 (Drosophila)
1284	COL4A2	collagen, type IV, alpha 2
1286	COL4A4	collagen, type IV, alpha 4
1288	COL4A6	collagen, type IV, alpha 6
1287	COL4A5	collagen, type IV, alpha 5
1282	COL4A1	collagen, type IV, alpha 1
284217	LAMA1	laminin, alpha 1
3908	LAMA2	laminin, alpha 2
3911	LAMA5	laminin, alpha 5
3909	LAMA3	laminin, alpha 3
3910	LAMA4	laminin, alpha 4
3912	LAMB1	laminin, beta 1
3913	LAMB2	laminin, beta 2 (laminin S)
3914	LAMB3	laminin, beta 3
22798	LAMB4	laminin, beta 4
3915	LAMC1	laminin, gamma 1 (formerly LAMB2)
3918	LAMC2	laminin, gamma 2
10319	LAMC3	laminin, gamma 3
2335	FN1	fibronectin 1
3673	ITGA2	integrin, alpha 2 (CD49B, alpha 2 subunit of VLA-2 receptor)
3674	ITGA2B	integrin, alpha 2b (platelet glycoprotein IIb of IIb/IIIa complex, antigen CD41)
3675	ITGA3	integrin, alpha 3 (antigen CD49C, alpha 3 subunit of VLA-3 receptor)

3655	ITGA6	integrin, alpha 6
3685	ITGAV	integrin, alpha V (vitronectin receptor, alpha polypeptide, antigen CD51)
3688	ITGB1	integrin, beta 1 (fibronectin receptor, beta polypeptide, antigen CD29 includes MDF2, MSK12)
5747	PTK2	PTK2 protein tyrosine kinase 2 (EC:2.7.10.2)
5293	PIK3CD	phosphoinositide-3-kinase, catalytic, delta polypeptide (EC:2.7.1.153)
5291	PIK3CB	phosphoinositide-3-kinase, catalytic, beta polypeptide (EC:2.7.1.153)
5294	PIK3CG	phosphoinositide-3-kinase, catalytic, gamma polypeptide (EC:2.7.11.1 2.7.1.153)
5290	PIK3CA	phosphoinositide-3-kinase, catalytic, alpha polypeptide (EC:2.7.11.1 2.7.1.153)
8503	PIK3R3	phosphoinositide-3-kinase, regulatory subunit 3 (gamma)
23533	PIK3R5	phosphoinositide-3-kinase, regulatory subunit 5
5296	PIK3R2	phosphoinositide-3-kinase, regulatory subunit 2 (beta)
5295	PIK3R1	phosphoinositide-3-kinase, regulatory subunit 1 (alpha)
5728	PTEN	phosphatase and tensin homolog (EC:3.1.3.67 3.1.3.16 3.1.3.48)
4824	NKX3-1	NK3 homeobox 1
207	AKT1	v-akt murine thymoma viral oncogene homolog 1 (EC:2.7.11.1)
208	AKT2	v-akt murine thymoma viral oncogene homolog 2 (EC:2.7.11.1)
10000	AKT3	v-akt murine thymoma viral oncogene homolog 3 (protein kinase B, gamma) (EC:2.7.11.1)
1147	CHUK	conserved helix-loop-helix ubiquitous kinase (EC:2.7.11.10)
3551	IKBKB	inhibitor of kappa light polypeptide gene enhancer in B-cells, kinase beta (EC:2.7.11.10)
8517	IKBKG	inhibitor of kappa light polypeptide gene enhancer in B-cells, kinase gamma
4792	NFKBIA	nuclear factor of kappa light polypeptide gene enhancer in B-cells inhibitor, alpha
4790	NFKB1	nuclear factor of kappa light polypeptide gene enhancer in B-cells 1
4791	NFKB2	nuclear factor of kappa light polypeptide gene enhancer in B-cells 2 (p49/p100)
5970	RELA	v-rel reticuloendotheliosis viral oncogene homolog A (avian)
5743	PTGS2	prostaglandin-endoperoxide synthase 2 (prostaglandin G/H synthase and cyclooxygenase) (EC:1.14.99.1)
4843	NOS2	nitric oxide synthase 2, inducible (EC:1.14.13.39)

596	BCL2	B-cell CLL/lymphoma 2
330	BIRC3	baculoviral IAP repeat containing 3
331	XIAP	X-linked inhibitor of apoptosis
329	BIRC2	baculoviral IAP repeat containing 2
598	BCL2L1	BCL2-like 1
7185	TRAF1	TNF receptor-associated factor 1
7186	TRAF2	TNF receptor-associated factor 2
7187	TRAF3	TNF receptor-associated factor 3
9618	TRAF4	TNF receptor-associated factor 4
7188	TRAF5	TNF receptor-associated factor 5
7189	TRAF6	TNF receptor-associated factor 6, E3 ubiquitin protein ligase
2475	MTOR	mechanistic target of rapamycin (serine/threonine kinase) (EC:2.7.11.1)
572	BAD	BCL2-associated agonist of cell death
2308	FOXO1	forkhead box O1
4193	MDM2	Mdm2, p53 E3 ubiquitin protein ligase homolog (mouse) (EC:6.3.2.19)
7157	TP53	tumor protein p53
1027	CDKN1B	cyclin-dependent kinase inhibitor 1B (p27, Kip1)
1026	CDKN1A	cyclin-dependent kinase inhibitor 1A (p21, Cip1)
613	BCR	breakpoint cluster region (EC:2.7.11.1)
25	ABL1	c-abl oncogene 1, non-receptor tyrosine kinase (EC:2.7.10.2)
1399	CRKL	v-crk sarcoma virus CT10 oncogene homolog (avian)-like
1398	CRK	v-crk sarcoma virus CT10 oncogene homolog (avian)
23624	CBLC	Cbl proto-oncogene, E3 ubiquitin protein ligase C (EC:6.3.2.19)
868	CBLB	Cbl proto-oncogene, E3 ubiquitin protein ligase B (EC:6.3.2.19)
867	CBL	Cbl proto-oncogene, E3 ubiquitin protein ligase (EC:6.3.2.19)
6776	STAT5A	signal transducer and activator of transcription 5A
6777	STAT5B	signal transducer and activator of transcription 5B
3716	JAK1	Janus kinase 1 (EC:2.7.10.2)
6774	STAT3	signal transducer and activator of transcription 3 (acute-phase response factor)
6772	STAT1	signal transducer and activator of transcription 1, 91kDa
7423	VEGFB	vascular endothelial growth factor B
5228	PGF	placental growth factor
7422	VEGFA	vascular endothelial growth factor A
7424	VEGFC	vascular endothelial growth factor C
2277	FIGF	c-fos induced growth factor (vascular endothelial growth factor D)

7039	TGFA	transforming growth factor, alpha
1950	EGF	epidermal growth factor
1956	EGFR	epidermal growth factor receptor (EC:2.7.10.1)
2064	ERBB2	v-erb-b2 erythroblastic leukemia viral oncogene homolog 2, neuro/glioblastoma derived oncogene homolog (avian) (EC:2.7.10.1)
5154	PDGFA	platelet-derived growth factor alpha polypeptide
5155	PDGFB	platelet-derived growth factor beta polypeptide
5156	PDGFRA	platelet-derived growth factor receptor, alpha polypeptide (EC:2.7.10.1)
5159	PDGFRB	platelet-derived growth factor receptor, beta polypeptide (EC:2.7.10.1)
3479	IGF1	insulin-like growth factor 1 (somatomedin C)
3480	IGF1R	insulin-like growth factor 1 receptor (EC:2.7.10.1)
4254	KITLG	KIT ligand
3815	KIT	v-kit Hardy-Zuckerman 4 feline sarcoma viral oncogene homolog (EC:2.7.10.1)
2323	FLT3LG	fms-related tyrosine kinase 3 ligand
2322	FLT3	fms-related tyrosine kinase 3 (EC:2.7.10.1)
3082	HGF	hepatocyte growth factor (hepapoietin A
4233	MET	met proto-oncogene (hepatocyte growth factor receptor) (EC:2.7.10.1)
2258	FGF13	fibroblast growth factor 13
2253	FGF8	fibroblast growth factor 8 (androgen-induced)
8817	FGF18	fibroblast growth factor 18
2257	FGF12	fibroblast growth factor 12
2248	FGF3	fibroblast growth factor 3
2251	FGF6	fibroblast growth factor 6
2256	FGF11	fibroblast growth factor 11
8823	FGF16	fibroblast growth factor 16
2252	FGF7	fibroblast growth factor 7
2259	FGF14	fibroblast growth factor 14
8822	FGF17	fibroblast growth factor 17
9965	FGF19	fibroblast growth factor 19
2254	FGF9	fibroblast growth factor 9 (glia-activating factor)
2250	FGF5	fibroblast growth factor 5
2249	FGF4	fibroblast growth factor 4
8074	FGF23	fibroblast growth factor 23
27006	FGF22	fibroblast growth factor 22
26281	FGF20	fibroblast growth factor 20
2255	FGF10	fibroblast growth factor 10
2247	FGF2	fibroblast growth factor 2 (basic)

26291	FGF21	fibroblast growth factor 21
2246	FGF1	fibroblast growth factor 1 (acidic)
2260	FGFR1	fibroblast growth factor receptor 1 (EC:2.7.10.1)
2263	FGFR2	fibroblast growth factor receptor 2 (EC:2.7.10.1)
2261	FGFR3	fibroblast growth factor receptor 3 (EC:2.7.10.1)
2885	GRB2	growth factor receptor-bound protein 2
6654	SOS1	son of sevenless homolog 1 (Drosophila)
6655	SOS2	son of sevenless homolog 2 (Drosophila)
3265	HRAS	v-Ha-ras Harvey rat sarcoma viral oncogene homolog
3845	KRAS	v-Ki-ras2 Kirsten rat sarcoma viral oncogene homolog
4893	NRAS	neuroblastoma RAS viral (v-ras) oncogene homolog
369	ARAF	v-raf murine sarcoma 3611 viral oncogene homolog (EC:2.7.11.1)
673	BRAF	v-raf murine sarcoma viral oncogene homolog B1 (EC:2.7.11.1)
5894	RAF1	v-raf-1 murine leukemia viral oncogene homolog 1 (EC:2.7.11.1)
5604	MAP2K1	mitogen-activated protein kinase kinase 1 (EC:2.7.12.2)
5605	MAP2K2	mitogen-activated protein kinase kinase 2 (EC:2.7.12.2)
5594	MAPK1	mitogen-activated protein kinase 1 (EC:2.7.11.24)
5595	MAPK3	mitogen-activated protein kinase 3 (EC:2.7.11.24)
3725	JUN	jun proto-oncogene
2353	FOS	FBJ murine osteosarcoma viral oncogene homolog
4312	MMP1	matrix metallopeptidase 1 (interstitial collagenase) (EC:3.4.24.7)
4313	MMP2	matrix metallopeptidase 2 (gelatinase A, 72kDa gelatinase, 72kDa type IV collagenase) (EC:3.4.24.24)
4318	MMP9	matrix metallopeptidase 9 (gelatinase B, 92kDa gelatinase, 92kDa type IV collagenase) (EC:3.4.24.35)
3576	IL8	interleukin 8
1019	CDK4	cyclin-dependent kinase 4 (EC:2.7.11.22)
5979	RET	ret proto-oncogene (EC:2.7.10.1)
8030	CCDC6	coiled-coil domain containing 6
8031	NCOA4	nuclear receptor coactivator 4
4914	NTRK1	neurotrophic tyrosine kinase, receptor, type 1 (EC:2.7.10.1)
7170	TPM3	tropomyosin 3
7175	TPR	translocated promoter region (to activated MET oncogene)
10342	TFG	TRK-fused gene
11186	RASSF1	Ras association (RalGDS/AF-6) domain family member 1
83593	RASSF5	Ras association (RalGDS/AF-6) domain family member 5
6789	STK4	serine/threonine kinase 4 (EC:2.7.11.1)
1613	DAPK3	death-associated protein kinase 3 (EC:2.7.11.1)

23604	DAPK2	death-associated protein kinase 2 (EC:2.7.11.1)
1612	DAPK1	death-associated protein kinase 1 (EC:2.7.11.1)
5335	PLCG1	phospholipase C, gamma 1 (EC:3.1.4.11)
5336	PLCG2	phospholipase C, gamma 2 (phosphatidylinositol-specific) (EC:3.1.4.11)
5579	PRKCB	protein kinase C, beta (EC:2.7.11.13)
5578	PRKCA	protein kinase C, alpha (EC:2.7.11.13)
5582	PRKCG	protein kinase C, gamma (EC:2.7.11.13)
5900	RALGDS	ral guanine nucleotide dissociation stimulator
5898	RALA	v-ral simian leukemia viral oncogene homolog A (ras related)
5899	RALB	v-ral simian leukemia viral oncogene homolog B (ras related
10928	RALBP1	ralA binding protein 1
998	CDC42	cell division cycle 42 (GTP binding protein, 25kDa)
5879	RAC1	ras-related C3 botulinum toxin substrate 1 (rho family, small GTP binding protein Rac1)
5880	RAC2	ras-related C3 botulinum toxin substrate 2 (rho family, small GTP binding protein Rac2)
5881	RAC3	ras-related C3 botulinum toxin substrate 3 (rho family, small GTP binding protein Rac3)
387	RHOA	ras homolog family member A
5602	MAPK10	mitogen-activated protein kinase 10 (EC:2.7.11.24)
5601	MAPK9	mitogen-activated protein kinase 9 (EC:2.7.11.24)
5599	MAPK8	mitogen-activated protein kinase 8 (EC:2.7.11.24)
7849	PAX8	paired box 8
5468	PPARG	peroxisome proliferator-activated receptor gamma
6256	RXRA	retinoid X receptor, alpha
6257	RXRB	retinoid X receptor, beta
6258	RXRG	retinoid X receptor, gamma
5915	RARB	retinoic acid receptor, beta
5467	PPARD	peroxisome proliferator-activated receptor delta
3728	JUP	junction plakoglobin
7704	ZBTB16	zinc finger and BTB domain containing 16
5371	PML	promyelocytic leukemia
5914	RARA	retinoic acid receptor, alpha
861	RUNX1	runt-related transcription factor 1
862	RUNX1T1	runt-related transcription factor 1
6688	SPI1	spleen focus forming virus (SFFV) proviral integration oncogene spi1
1050	CEBPA	CCAAT/enhancer binding protein (C/EBP), alpha
1438	CSF2RA	colony stimulating factor 2 receptor, alpha, low-affinity (granulocyte-macrophage)
1441	CSF3R	colony stimulating factor 3 receptor (granulocyte)

1436	CSF1R	colony stimulating factor 1 receptor (EC:2.7.10.1)
3569	IL6	interleukin 6 (interferon, beta 2)
1029	CDKN2A	cyclin-dependent kinase inhibitor 2A (melanoma, p16, inhibits CDK4)
1871	E2F3	E2F transcription factor 3
1869	E2F1	E2F transcription factor 1
1870	E2F2	E2F transcription factor 2
4149	MAX	MYC associated factor X
8554	PIAS1	protein inhibitor of activated STAT, 1
51588	PIAS4	protein inhibitor of activated STAT, 4
10401	PIAS3	protein inhibitor of activated STAT, 3
9063	PIAS2	protein inhibitor of activated STAT, 2
1030	CDKN2B	cyclin-dependent kinase inhibitor 2B (p15, inhibits CDK4)
1021	CDK6	cyclin-dependent kinase 6 (EC:2.7.11.22)
1163	CKS1B	CDC28 protein kinase regulatory subunit 1B
6502	SKP2	S-phase kinase-associated protein 2, E3 ubiquitin protein ligase
1017	CDK2	cyclin-dependent kinase 2 (EC:2.7.11.22)
9134	CCNE2	cyclin E2
898	CCNE1	cyclin E1
5925	RB1	retinoblastoma 1
4286	MITF	microphthalmia-associated transcription factor
7040	TGFB1	transforming growth factor, beta 1
7042	TGFB2	transforming growth factor, beta 2
7043	TGFB3	transforming growth factor, beta 3
7046	TGFBR1	transforming growth factor, beta receptor 1 (EC:2.7.11.30)
7048	TGFBR2	transforming growth factor, beta receptor II (70/80kDa) (EC:2.7.11.30)
4087	SMAD2	SMAD family member 2
4088	SMAD3	SMAD family member 3
4089	SMAD4	SMAD family member 4
2122	MECOM	MDS1 and EVI1 complex locus
1488	CTBP2	C-terminal binding protein 2
1487	CTBP1	C-terminal binding protein 1
3066	HDAC2	histone deacetylase 2 (EC:3.5.1.98)
3065	HDAC1	histone deacetylase 1 (EC:3.5.1.98)
4292	MLH1	mutL homolog 1, colon cancer, nonpolyposis type 2 (E. coli)
4436	MSH2	mutS homolog 2, colon cancer, nonpolyposis type 1 (E. coli)
4437	MSH3	mutS homolog 3 (E. coli)
2956	MSH6	mutS homolog 6 (E. coli)
581	BAX	BCL2-associated X protein
675	BRCA2	breast cancer 2, early onset

5888	RAD51	RAD51 homolog (S. cerevisiae)
356	FASLG	Fas ligand (TNF superfamily, member 6)
355	FAS	Fas (TNF receptor superfamily, member 6)
8772	FADD	Fas (TNFRSF6)-associated via death domain
841	CASP8	caspase 8, apoptosis-related cysteine peptidase (EC:3.4.22.61)
637	BID	BH3 interacting domain death agonist
54205	CYCS	cytochrome c, somatic
7428	VHL	von Hippel-Lindau tumor suppressor, E3 ubiquitin protein ligase
6921	TCEB1	transcription elongation factor B (SIII), polypeptide 1 (15kDa, elongin C)
6923	TCEB2	transcription elongation factor B (SIII), polypeptide 2 (18kDa, elongin B)
9978	RBX1	ring-box 1, E3 ubiquitin protein ligase
8453	CUL2	cullin 2
112399	EGLN3	egl nine homolog 3 (C. elegans) (EC:1.14.11.29)
112398	EGLN2	egl nine homolog 2 (C. elegans) (EC:1.14.11.29)
54583	EGLN1	egl nine homolog 1 (C. elegans) (EC:1.14.11.29)
2271	FH	fumarate hydratase (EC:4.2.1.2)
3091	HIF1A	hypoxia inducible factor 1, alpha subunit (basic helix-loop-helix transcription factor)
2034	EPAS1	endothelial PAS domain protein 1
405	ARNT	aryl hydrocarbon receptor nuclear translocator
9915	ARNT2	aryl-hydrocarbon receptor nuclear translocator 2
1387	CREBBP	CREB binding protein (EC:2.3.1.48)
2033	EP300	E1A binding protein p300 (EC:2.3.1.48)
6513	SLC2A1	solute carrier family 2 (facilitated glucose transporter), member 1
6469	SHH	sonic hedgehog
5727	PTCH1	patched 1
6608	SMO	smoothened, frizzled family receptor
27148	STK36	serine/threonine kinase 36 (EC:2.7.11.1)
51684	SUFU	suppressor of fused homolog (Drosophila)
2737	GLI3	GLI family zinc finger 3
2736	GLI2	GLI family zinc finger 2
2735	GLI1	GLI family zinc finger 1
650	BMP2	bone morphogenetic protein 2
652	BMP4	bone morphogenetic protein 4
64399	HHIP	hedgehog interacting protein
8643	PTCH2	patched 2
367	AR	androgen receptor

3320	HSP90AA1	heat shock protein 90kDa alpha (cytosolic), class A member 1
3326	HSP90AB1	heat shock protein 90kDa alpha (cytosolic), class B member 1
7184	HSP90B1	heat shock protein 90kDa beta (Grp94), member 1
354	KLK3	kallikrein-related peptidase 3 (EC:3.4.21.77)
112401	BIRC8	baculoviral IAP repeat containing 8
2113	ETS1	v-ets erythroblastosis virus E26 oncogene homolog 1 (avian)
2950	GSTP1	glutathione S-transferase pi 1 (EC:2.5.1.18)
5337	PLD1	phospholipase D1, phosphatidylcholine-specific (EC:3.1.4.4)
79444	BIRC7	baculoviral IAP repeat containing 7
8900	CCNA1	cyclin A1
