# Supplementary material for: An Approach for the Identification of Targets Specific to Bone Metastasis Using Cancer Genes Interactome and Gene Ontology Analysis
Source: PLoS One. 2012 Nov 14;7(11):e49401. doi: 10.1371/journal.pone.0049401 (PMC3498148; doi:10.1371/journal.pone.0049401)
Supplement: Table S3 — SBCGs (391) compiled for metastasis of primary breast and prostate cancer to bone. (PDF) [file pone.0049401.s003.pdf]

Table S3. SBCGs (391) compiled for metastasis of primary breast and prostate cancer to bone.

SBCGs compiled for metastasis of primary breast cancer to bone	
**Gene Symbol**	**Reference**
LRRC15, MMP13, MMP2, BCAM, COL10A1, AEBP1, IGFBP4, ESR1, AEBP1, ASPN, ATP6V0C, BCAM, COMP, CRIP1, DPT, IGFBP4, LRRC15, LRP1, MAB21L2, MMP13, MMP2, PDLIM7, PTOV1, PCDHGC3, RFNG, STK32B, TBC1D10B, TCIRG1, ZNF444	[1]
COX2, PGE2, TNFSF11, TNFRSF11A, FGF7, PTX3, NID2, RAP1GDS1, NPNT, COL6A3, IFIT3, IGRC, FMOD, IGIT1B, EGR1, PTGS2, CXCL10, VCAM1, FGF7, PTN, CD1D, LAMB1, CCL5, SFRP2, CXCL1	[2]
RASSF1A, MGMT, RAR-β2, APC	[3]
IBSP, SPP1, FGF13, COX7B2, SPARC, PRG1, GJA1, SOCS2, EDN1, IL11, CYR61, IGFBP4, IL8, PLAU, FST, MADH3, PTGS2, EXTL2, CTGF, CBFB, CDH11, S100A4, TIMP3, NOG, TGFA, THBS1, IGFBP3, PLAT, TIMP2, FN1, CCNB1, LRPAP1, MMP1, COL5A1, COL8A1, CALB2, CYR61, CTGF, WISP, BMP2, IL11, FST, CXCR4, ADAMTS1, MMP1, CX43, DUSP1, NOG, COX2, CBFB, GJA1, THBS1	[4]
SERPINB5	[5]
ABCB1, ESR1, RUNDC3B, RAP2A	[6]
CSFIR	[7]
CAV1	[8]
TGFB1, ATF3, MMP13, CCNA1	[9]
COL1A1, VIM, S100A4, ACTA2, PDGFRB, NG2, CXCL1, CXCL5, PTGS2	[10]
HISTIH2AC, POMZP3, PON2, TMC5, SCNN1A, C10orf116, NAP1L3, GPRD5C, CTGF, FHL1, DUSP1, DLC1, SOCS2, IL11, ADAMTS1, CYP1B1, SAA2, SAA1, PPL, IL11, SPANXB1, FST, SERPINA1, HLA-DRA, HLA-DPA1, ABCC3, CSGALNACT1, FKBP11, CXCR4, PRG1, SES7-1, SOX4, KHDRBS3, FGF5, C14orf1139, PTX7, MCAM, TGFBI, S100A2, MMP1, FGF5, RGC32	[11]
TFF1, TFF3, AGR2, NAT1, CRIP1, TNRC9, SCUBE2, TNRC9, CYP2B6, RND1, DLALT1, KIF5C, PLA2GHB, UNG2, HMGCS2, SLC1A1, CEACAM6, TSPAN-1, REPS2, HPX, PDE4DIP, TOM1L1, SCGB2A2, ANXA9, BCAS1, TIMP4, CYP2B6, C9orf116, MSMB, FGFR3, FGFBP1, BAG1, FOXO3A, KRT16, MALL, KCNG1, IGLC2, KLK8, KRT6B, SNAI1, TMSNB, NRTN, EPHB3, ENO1, SOD2, IGHG1, KLK5, SOS, RARRES1, TTYH1, CD24, MGC27165, SERPINB5, UCHL1, ROPN1, LOC56901, ITGA6, TUBB, UBE4B, IGHM, COL2A1, IMPA2, DRE1, KLK7, TAZ, USP34, C6orf4, ALAD, ARHGDIA, ARS2, CLCA2, ELMO2, LRRC31, HTATSF1, HTR2B, MLPH, SLC2A8, TFPI2, BRCA1, BRCA2	[12]

DIP2B, MCM6, PLEK2 , FOXI1 , SYT13 , PRDM6 , LONP2, ZNF185, ENTPD4, PPP2R2C, LANCL2, SFTPD, TSGA10, B3GALT2, SEZ6L, KCND2, VSTM2, FANCL, FDPS , VRK2	[13]
HIF1A, VEGF, DUSP1, CXCR4	[14]
IL1B, COL1A1, COL1A2, CTGF, IL10, IL8, SPP1, VCAM1, PGE2, EDN1, BMP6, IL6, PDGFRA, PTHLH, TGFB, LAMC2, IGFBP3, PLAU, TNF, TNFSF11, TNFRSF11A, TNFRSF11B, CXCR4, CXCL12	[15]
CXCR4, CXCL12, HER2	[16]
PASAT1, PHGDH, PSPH, LTBP1, ATF3, CCNA1, MMP13	[17]
ADAM9, ADAMTS1, AREG, EGF, EGFR, ERBB2, HBEGF, MMP1, PTHLH, TNFRSF11A, TNFRSF11B	[18]
CTNNB1, DKK1, PLAU, PLAUR, SERPINB2, SERPINE1, WNT3A	[19]
BMP4, FGF1, FGF17, FGF4, FGF6, FGF8, IGF1, IL11, IL1B, IL6, IL8	[20]
CTTN, CXCL12, CXCR4, IL11, IL6,	[21]
ADAMTS1, EGF, EGFR, MMP1, TNFRSF11A, TNFRSF11B	[22]
HIF1A	[23]
AKT1, AKT3, BMP2, CLEC11A, CXCL12, CXCR4, JAG1, NOV, PDGFA, PGF, PRG2, SPP1, SRC, TGFB1, TGFB3, TNFSF10, VEGFC	[24]
CTGF, NOV, WISP2, WISP3	[25]
CSF1, IL8, PTHLH, TNFRSF11A, TNFRSF11B	[26]
**SBCGs compiled for metastasis of primary prostate cancer to bone**	
APX1, ANTXR1, CDC23, CDC37, CETN3, CENPE, CCNC, CCNG1, CDK4, CDKN2C, CDKN3, CDKL1, DDB1, E2F1, FEN1, GTPBP4, KAT7, MCM7, MAD2L1,MSH6, NBN, PCNA, EIF2AK2, RAN, RPA2, RPA3, RRM2, SSBP1, SKP1, ADD1, DST, CDH1, CTNNAL1, CYB5R3, DSC1, DSG2, ADAM9, FER, TNC, ITGB1, MFGE8, MCAM, ACVRL1, BGN, BST1, IBSP, CDH11, COL1A2, COL6A1, COL6A2, COL7A1, COL11A1, COL16A1, CSF1, DCN, FSTL1, INHA, MGP, SHH, VCAN, COL17A1, ITGB4, PLEC, DSC2, DSP, PKP1, PKP2, PKP3, PKP4, ASRGL1, BMP2, VDR, RUNX2, BGLAP, SPARC, SPP1, TNFRSF11B, TNFSF11, DRG1, PTHRP, EDN1, TNFRSF11A, TP53, HPN, TFF3, BGLAP, ERBB3, PSMD9, CDKN1A, MYCN, AR, HIF1A, MAGEA1, VEGF, KLK3, GDF15, BMP6, ERBB2, LTBP1, MMP9, MMP1, MMP3, PLAU, HRAS, NRAS, KRT1, PTRF, MDM2, RB1	[27]
HPN	[28]
TFF3	[29]
BGLAP	[30]
ERBB3	[31]
PSMD9, CDKN1A	[27]
ERBB2, EGFR	[32]
PALB, TGFB1, EGFR, BMP	[33]
BIN1, MYC, ABL1	[34]
MAGEA1	[35]

MMP1, MMP2, MMP13, DRG1, PTEN, NME1, CD82, KISS1, BRMS1, MAP2K4, TP53	[36]
IGFBP3, PTH1R, TNFSF11, MMP, MDM2, RB1, TNFRSF11A	[37]
RB1	[38]
CDH11	[39]
LYN, SRC	[40]
ADAMTS1, AREG, EGF, EGFR, HBEGF, PTHLH, TGFA, TNF, TNFRSF11A, TNFRSF11B, VEGFA	[18]
VEGFA, NR1I2	[41]
AKT1, PTHLH, TNFRSF11A, TNFRSF11B	[42]
BMP4, CSF1, DKK1, IL8, NOG, PTHLH, SFRP1, SFRP2, TGFB1, TNFRSF11A, TNFRSF11B	[43]
RAP1GAP, TERF2IP, VTNR	[44]
CXCL12, CXCR4, MAPK1, MAPK3,	[45]
FGF1, FGF17, FGF4, FGF6, FGF8, FGFR3,	[46]

References

1. Klein A, Olendrowitz C, Schmutzler R, Hampl J, Schlag PM, et al. (2009) Identification of brain- and bone-specific breast cancer metastasis genes. Cancer letters 276: 212–220. doi:10.1016/j.canlet.2008.11.017.

2. Li Z, Schem C, Shi YH, Medina D, Zhang M (2008) Increased COX2 expression enhances tumor-induced osteoclastic lesions in breast cancer bone metastasis. Clinical & experimental metastasis 25: 389–400. doi:10.1007/s10585-007-9117-3.

3. Taback B, Giuliano AE, Lai R, Hansen N, Singer FR, et al. (2006) Epigenetic analysis of body fluids and tumor tissues: application of a comprehensive molecular assessment for early-stage breast cancer patients. Annals of the New York Academy of Sciences 1075: 211–221. doi:10.1196/annals.1368.029.

4. Bellahcène A, Bachelier R, Detry C, Lidereau R, Clézardin P, et al. (2007) Transcriptome analysis reveals an osteoblast-like phenotype for human osteotropic breast cancer cells. Breast cancer research and treatment 101: 135–148. doi:10.1007/s10549-006-9279-8.

5. Maass N, Hojo T, Rösel F, Ikeda T, Jonat W, et al. (2001) Down regulation of the tumor suppressor gene maspin in breast carcinoma is associated with a higher risk of distant metastasis. Clinical biochemistry 34: 303–307.

6. Raguz S, De Bella MT, Slade MJ, Higgins CF, Coombes RC, et al. (2005) Expression of RPIP9 (Rap2 interacting protein 9) is activated in breast carcinoma and correlates with a poor prognosis. International journal of cancer 117: 934–941. doi:10.1002/ijc.21252.

7. Storga D, Pećina-Slaus N, Pavelić J, Pavelić ZP, Pavelić K (1992) C-Fms is present in primary tumours as well as in their metastases in bone marrow. International journal of experimental pathology 73: 527–533.

8. Sloan EK, Stanley KL, Anderson RL (2004) Caveolin-1 inhibits breast cancer growth and metastasis. Oncogene 23: 7893–7897. doi:10.1038/sj.onc.1208062.

9. Kwok S, Rittling SR, Partridge NC, Benson CS, Thiyagaraj M, et al. (2009) Transforming growth factor-beta1 regulation of ATF-3 and identification of ATF-3 target genes in breast cancer cells. Journal of cellular biochemistry 108: 408–414. doi:10.1002/jcb.22267.

10. Bierie B, Stover DG, Abel TW, Chytil A, Gorska AE, et al. (2008) Transforming growth factor-beta regulates mammary carcinoma cell survival and interaction with the adjacent microenvironment. Cancer research 68: 1809–1819. doi:10.1158/0008-5472.CAN-07-5597.

11. Minn AJ, Kang Y, Serganova I, Gupta GP, Giri DD, et al. (2005) Distinct organ-specific metastatic potential of individual breast cancer cells and primary tumors. The Journal of Clinical Investigation 115: 44–55. doi:10.1172/JCI200522320.44.

12. Smid M, Wang Y, Klijn JGM, Sieuwerts AM, Zhang Y, et al. (2006) Genes associated with breast cancer metastatic to bone. Journal of clinical oncology 24: 2261–2267. doi:10.1200/JCO.2005.03.8802.

13. Naume B, Zhao X, Synnestvedt M, Borgen E, Russnes HG, et al. (2007) Presence of bone marrow micrometastasis is associated with different recurrence risk within molecular subtypes of breast cancer. Molecular oncology 1: 160–171. doi:10.1016/j.molonc.2007.03.004.

14. Lu X, Yan CH, Yuan M, Wei Y, Hu G, et al. (2010) In vivo dynamics and distinct functions of hypoxia in primary tumor growth and organotropic metastasis of breast cancer. Cancer research 70: 3905–3914. doi:10.1158/0008-5472.CAN-09-3739.

15. Yin JJ, Pollock CB, Kelly K (2005) Mechanisms of cancer metastasis to the bone. Cell research 15: 57–62. doi:10.1038/sj.cr.7290266.

16. Sacanna E, Ibrahim T, Gaudio M, Mercatali L, Scarpi E, et al. (2011) The role of CXCR4 in the prediction of bone metastases from breast cancer: a pilot study. Oncology 80: 225–231.

17. Pollari S, Käkönen S-M, Edgren H, Wolf M, Kohonen P, et al. (2011) Enhanced serine production by bone metastatic breast cancer cells stimulates osteoclastogenesis. Breast cancer research and treatment 125: 421–430. doi:10.1007/s10549-010-0848-5.

18. Lu X, Wang Q, Hu G, Van Poznak C, Fleisher M, et al. (2009) ADAMTS1 and MMP1 proteolytically engage EGF-like ligands in an osteolytic signaling cascade for bone metastasis. Genes & Development 23: 1882–1894. doi:10.1101/gad.1824809.

19. Bu G, Lu W, Liu C-C, Selander K, Yoneda T, et al. (2008) Breast cancer-derived Dickkopf1 inhibits osteoblast differentiation and osteoprotegerin expression: implication for breast cancer osteolytic bone metastases. International Journal of Cancer 123: 1034–1042. doi:10.1002/ijc.23625.

20. Kozlow W, Guise TA (2005) Breast cancer metastasis to bone: mechanisms of osteolysis and implications for therapy. Journal of Mammary Gland Biology and Neoplasia 10: 169–180. doi:10.1007/s10911-005-5399-8.

21. Weigelt B, Peterse J (2005) Breast cancer metastasis: markers and models. Nature reviews cancer 5: 591–602. doi:10.1038/nrc1670.

22. Guise TA (2009) Breaking down bone: new insight into site-specific mechanisms of breast cancer osteolysis mediated by metalloproteinases. Genes & Development 23: 2117–2123. doi:10.1101/gad.1854909.

23. Hiraga T, Kizaka-Kondoh S, Hirota K, Hiraoka M, Yoneda T (2007) Hypoxia and hypoxia-inducible factor-1 expression enhance osteolytic bone metastases of breast cancer. Cancer Research 67: 4157–4163. doi:10.1158/0008-5472.CAN-06-2355.

24. Zhang XH-F, Wang Q, Gerald W, Hudis C a, Norton L, et al. (2009) Latent bone metastasis in breast cancer tied to Src-dependent survival signals. Cancer Cell 16: 67–78. doi:10.1016/j.ccr.2009.05.017.

25. Shimo T, Kubota S, Yoshioka N, Ibaragi S, Isowa S, et al. (2006) Pathogenic Role of Connective Tissue Growth Factor (CTGF/CCN2) in Osteolytic Metastasis of Breast Cancer. Journal of Bone and Mineral Research 21: 1045–1059. doi:10.1359/JBMR.060416.

26. Cicek M, Oursler MJ (2006) Breast cancer bone metastasis and current small therapeutics. Cancer Metastasis Reviews 25: 635–644. doi:10.1007/s10555-006-9035-x.

27. Knerr K, Ackermann K, Neidhart T, Pyerin W (2004) Bone metastasis: Osteoblasts affect growth and adhesion regulons in prostate tumor cells and provoke osteomimicry. International journal of cancer 111: 152–159. doi:10.1002/ijc.20223.

28. Vasioukhin V (2004) Hepsin Paradox Reveals Unexpected Complexity of Metastatic Process. Cell Cycle 3: 1394–1397.

29. Faith D a, Isaacs WB, Morgan JD, Fedor HL, Hicks JL, et al. (2004) Trefoil factor 3 overexpression in prostatic carcinoma: prognostic importance using tissue microarrays. The Prostate 61: 215–227. doi:10.1002/pros.20095.

30. Chung LWK, Hsieh CL, Law A, Sung SY, Gardner TA, et al. (2003) New targets for therapy in prostate cancer: modulation of stromal-epithelial interactions. Urology 62: 44–54.

31. Vakar-Lopez F, Cheng C-J, Kim J, Shi GG, Troncoso P, et al. (2004) Up-regulation of MDA-BF-1, a secreted isoform of ErbB3, in metastatic prostate cancer cells and activated osteoblasts in bone marrow. The Journal of pathology 203: 688–695.

32. Morote J, de Torres I, Caceres C, Vallejo C, Schwartz S, et al. (1999) Prognostic value of immunohistochemical expression of the c-erbB-2 oncoprotein in metastasic prostate cancer. International Journal of Cancer 84: 421–425.

33. Thomas R, True LD, Lange PH, Vessella RL (2001) Placental bone morphogenetic protein (PLAB) gene expression in normal, pre-malignant and malignant human prostate: relation to tumor development and progression. International Journal of Cancer 93: 47–52.

34. Tamada H, Kitazawa R, Gohji K, Kitazawa S (2001) Epigenetic regulation of human bone morphogenetic protein 6 gene expression in prostate cancer. Journal of bone and mineral research 16: 487–496. doi:10.1359/jbmr.2001.16.3.487.

35. Kufer P, Zippelius A, Lutterbüse R, Mecklenburg I, Enzmann T, et al. (2002) Heterogeneous Expression of MAGE-A Genes in Occult Disseminated Tumor Cells : A Novel Multimarker Reverse Transcription-Polymerase Chain Reaction for Diagnosis of Micrometastatic Disease. Cancer research 62: 251–261.

36. Bandyopadhyay S, Pai SK, Gross SC, Hirota S, Hosobe S, et al. (2003) The Drg-1 Gene Suppresses Tumor Metastasis in Prostate Cancer. Cancer research 63: 1731–1736.

37. Fizazi K, Yang J, Peleg S, Sikes CR, Kreimann EL, et al. (2003) Prostate Cancer Cells-Osteoblast Interaction Shifts Expression of Growth / Survival-related Genes in Prostate Cancer and Reduces Expression of Osteoprotegerin in Osteoblasts. Cllinical cancer research 9: 2587–2597.

38. Bookstein R, Rio P, Madreperla S a, Hong F, Allred C, et al. (1990) Promoter deletion and loss of retinoblastoma gene expression in human prostate carcinoma. Proceedings of the National Academy of Sciences of the United States of America 87: 7762–7766.

39. Chu K, Cheng C-J, Ye X, Lee Y-C, Zurita AJ, et al. (2008) Cadherin-11 promotes the metastasis of prostate cancer cells to bone. Molecular Cancer Research 6: 1259–1267. doi:10.1158/1541-7786.MCR-08-0077.

40. Koreckij T, Nguyen H, Brown LG, Yu EY, Vessella RL, et al. (2009) Dasatinib inhibits the growth of prostate cancer in bone and provides additional protection from osteolysis. British Journal of Cancer 101: 263–268. doi:10.1038/sj.bjc.6605178.

41. Zhang X, Wang W, True LD, Vessella RL, Takayama TK (2009) Protease-activated receptor-1 is upregulated in reactive stroma of primary prostate cancer and bone metastasis. The Prostate 69: 727–736. doi:10.1002/pros.20920.

42. Jones DH, Nakashima T, Sanchez OH, Kozieradzki I, Komarova SV, et al. (2006) Regulation of cancer cell migration and bone metastasis by RANKL. Nature 440: 692–696. doi:10.1038/nature04524.

43. Secondini C, Wetterwald A, Schwaninger R, Thalmann GN, Cecchini MG (2011) The role of the BMP signaling antagonist noggin in the development of prostate cancer osteolytic bone metastasis. PloS One 6: e16078. doi:10.1371/journal.pone.0016078.

44. Bailey CL, Kelly P, Casey PJ (2009) Activation of Rap1 promotes prostate cancer metastasis. Cancer Research 69: 4962–4968. doi:10.1158/0008-5472.CAN-08-4269.

45. Taichman RS, Cooper C, Keller ET, Pienta KJ, Taichman NS, et al. (2002) Use of the Stromal Cell-derived Factor-1 / CXCR4 Pathway in Prostate Cancer Metastasis to Bone. Cancer Research 62: 1832–1837.

46. Valta MP, Tuomela J, Bjartell A, Valve E, Väänänen HK, et al. (2008) FGF-8 is involved in bone metastasis of prostate cancer. International Journal of Cancer 123: 22–31. doi:10.1002/ijc.23422.
