# Supplementary material for: An Approach for the Identification of Targets Specific to Bone Metastasis Using Cancer Genes Interactome and Gene Ontology Analysis
Source: PLoS One. 2012 Nov 14;7(11):e49401. doi: 10.1371/journal.pone.0049401 (PMC3498148; doi:10.1371/journal.pone.0049401)
Supplement: Table S4 — Significantly enriched GO terms, characteristic to metastasis to bone, identified from enrichment analysis of SBCGs. (PDF) [file pone.0049401.s004.pdf]

Table S4. Significantly enriched GO terms, characteristic to metastasis to bone, identified from enrichment analysis of SBCGs.

GO ID	GO terms	Reference
**Bone related processes**		
GO:0001958	endochondral ossification	[1]
GO:0036075	replacement ossification	[1]
GO:0030282	bone mineralization	[1]
GO:0030199	collagen fibril organization	[2]
GO:0032963	collagen metabolic process	[2]
GO:0033280	response to vitamin D	[3]
GO:0046850	regulation of bone remodeling	[4]
GO:0001649	osteoblast differentiation	[1,4]
GO:0001503	ossification	[1]
GO:0051216	cartilage development	[1]
**Metastasis related processes**		
GO:0002690	positive regulation of leukocyte chemotaxis	[5]
GO:0002688	regulation of leukocyte chemotaxis	[5]
GO:0002687	positive regulation of leukocyte migration	[5]
GO:0050920	regulation of chemotaxis	[5]
GO:0007162	negative regulation of cell adhesion	[6]
GO:0002685	regulation of leukocyte migration	[7]
GO:0050900	leukocyte migration	[7]
GO:0016337	cell-cell adhesion	[6,8]
GO:0001525	angiogenesis	[9,10]
GO:0050679	positive regulation of epithelial cell proliferation	[3]
GO:0045785	positive regulation of cell adhesion	[6]
GO:0043236	laminin binding	[11]
GO:0001968	fibronectin binding	[12,13]
GO:0005104	fibroblast growth factor receptor binding	[14]
GO:0048407	platelet-derived growth factor binding	[15,16]
GO:0005518	collagen binding	[2,17]
GO:0005178	integrin binding	[12,18]
GO:0005539	glycosaminoglycan binding	[19]
GO:0005125	cytokine activity	[20,21]
GO:0030246	carbohydrate binding	[22]
GO:0035413	positive regulation of catenin import into nucleus	[23]

References

1. Olszta MJ, Cheng X, Jee SS, Kumar R, Kim Y-Y, et al. (2007) Bone structure and formation: A new perspective. Materials Science and Engineering: R: Reports 58: 77–116. doi:10.1016/j.mser.2007.05.001.

2. Schönau E, Rauch F (1997) Markers of bone and collagen metabolism-problems and perspectives in paediatrics. Hormone research 48 Suppl 5: 50–59.

3. Sprenger CC, Peterson a, Lance R, Ware JL, Drivdahl RH, et al. (2001) Regulation of proliferation of prostate epithelial cells by 1,25-dihydroxyvitamin D3 is accompanied by an increase in insulin-like growth factor binding protein-3. The Journal of endocrinology 170: 609–618.

4. Andersen TL, Sondergaard TE, Skorzynska KE, Dagnaes-Hansen F, Plesner TL, et al. (2009) A physical mechanism for coupling bone resorption and formation in adult human bone. The American journal of pathology 174: 239–247. doi:10.2353/ajpath.2009.080627.

5. Moore M a (2001) The role of chemoattraction in cancer metastases. BioEssays 23: 674–676. doi:10.1002/bies.1095.

6. Hirohashi S, Kanai Y (2003) Cell adhesion system and human cancer morphogenesis. Cancer science 94: 575–581.

7. JONES BM (1976) MECHANISMS OF LEUCOCYTE MIGRATION INHIBITION BY BREAST TUMOUR CELL FRACTIONS. Br J Cancer 34: 14–19.

8. Okegawa T, Pong R-C, Li Y, Hsieh J-T (2004) The role of cell adhesion molecule in cancer progression and its application in cancer therapy. Acta biochimica Polonica 51: 445–457. doi:035001445.

9. Liotta LA, Steeg PS, Stetler-Stevenson WG (1991) Cancer metastasis and angiogenesis: An imbalance of positive and negative regulation. Cell 64: 327–336. doi:10.1016/0092-8674(91)90642-C.

10. Carmeliet P, Jain RK (2000) Angiogenesis in cancer and other diseases. Nature 407: 249–257.

11. Terranova VP, Llotta LA, Russo RG, Liotta LA, Martin GR (1982) Role of Laminin in the Attachment and Metastasis of Murine Tumor Cells Role of Laminin in the Attachment and Metastasis of Murine Tumor Cells. Cancer research 42: 2265–2269.

12. Akiyama SK, Olden K, Yamada KM (1995) Fibronectin and integrins in invasion and metastasis. Cancer and Metastasis Reviews 14: 173–189. doi:10.1007/BF00690290.

13. Malik G, Knowles LM, Dhir R, Xu S, Yang S, et al. (2010) Plasma fibronectin promotes lung metastasis by contributions to fibrin clots and tumor cell invasion. Cancer research 70: 4327–4334. doi:10.1158/0008-5472.CAN-09-3312.

14. Kwabi-Addo B, Ozen M, Ittmann M (2004) The role of fibroblast growth factors and their receptors in prostate cancer. Endocrine-related cancer 11: 709–724. doi:10.1677/erc.1.00535.

15. Yu J, Ustach C, Kim H-RC (2003) Platelet-derived growth factor signaling and human cancer. Journal of biochemistry and molecular biology 36: 49–59.

16. Russell MR, Liu Q, Lei H, Kazlauskas A, Fatatis A (2010) The alpha-receptor for platelet-derived growth factor confers bone-metastatic potential to prostate cancer cells by ligand- and dimerization-independent mechanisms. Cancer research 70: 4195–4203. doi:10.1158/0008-5472.CAN-09-4712.

17. Viguet-Carrin S, Garnero P, Delmas PD (2006) The role of collagen in bone strength. Osteoporosis international 17: 319–336. doi:10.1007/s00198-005-2035-9.

18. Vogelmann R, Kreuser ED, Adler G, Lutz MP (1999) Integrin alpha6beta1 role in metastatic behavior of human pancreatic carcinoma cells. International journal of cancer 80: 791–795.

19. Yip GW, Smollich M, Götte M (2006) Therapeutic value of glycosaminoglycans in cancer. Molecular cancer therapeutics 5: 2139–2148. doi:10.1158/1535-7163.MCT-06-0082.

20. Koizumi K, Hojo S, Akashi T, Yasumoto K, Saiki I (2007) Chemokine receptors in cancer metastasis and cancer cell-derived chemokines in host immune response. Cancer science 98: 1652–1658. doi:10.1111/j.1349-7006.2007.00606.x.

21. Cheng X, Hung M-C (2009) Regulation of breast cancer metastasis by atypical chemokine receptors. Clinical cancer research 15: 2951–2953. doi:10.1158/1078-0432.CCR-09-0141.

22. Kannagi R, Izawa M, Koike T, Miyazaki K, Kimura N (2004) Carbohydrate-mediated cell adhesion in cancer metastasis and angiogenesis. Cancer science 95: 377–384.

23. Kau TR, Way JC, Silver PA (2004) Nuclear transport and cancer: from mechanism to intervention. Nature reviews Cancer 4: 106–117. doi:10.1038/nrc1274.
