# Supplementary material for: An Approach for the Identification of Targets Specific to Bone Metastasis Using Cancer Genes Interactome and Gene Ontology Analysis
Source: PLoS One. 2012 Nov 14;7(11):e49401. doi: 10.1371/journal.pone.0049401 (PMC3498148; doi:10.1371/journal.pone.0049401)
Supplement: Table S5 — Relevance of SBC targets. (PDF) [file pone.0049401.s005.pdf]

Table S5. Relevance of SBC Targets.

SBC Specific Targets	Reference
TNXB	[1]
SPP1	[2]
CTGF	[3–5]
BMP1	[6]
BMPR1A	[6]
CD44	[7]
VWF	[8]

References

1. Matsumoto K, Takayama N, Ohnishi J, Ohnishi E, Shirayoshi Y, et al. (2001) Tumour invasion and metastasis are promoted in mice deficient in tenascin-X. Genes to cells 6: 1101–1111.

2. Rangaswami H, Bulbule A, Kundu GC (2006) Osteopontin: role in cell signaling and cancer progression. Trends in cell biology 16: 79–87. doi:10.1016/j.tcb.2005.12.005.

3. Chen P-S, Wang M-Y, Wu S-N, Su J-L, Hong C-C, et al. (2007) CTGF enhances the motility of breast cancer cells via an integrin-alphavbeta3-ERK1/2-dependent S100A4-upregulated pathway. Journal of cell science 120: 2053–2065. doi:10.1242/jcs.03460.

4. Lau LF, Lam SC (1999) MINIREVIEW The CCN Family of Angiogenic Regulators : The Integrin Connection. Experimental cell research 248: 44–57.

5. Kang Y, Siegel PM, Shu W, Drobnjak M, Kakonen SM, et al. (2003) A multigenic program mediating breast cancer metastasis to bone. Cancer cell 3: 537–549.

6. Mishina Y, Starbuck MW, Gentile M a, Fukuda T, Kasparcova V, et al. (2004) Bone morphogenetic protein type IA receptor signaling regulates postnatal osteoblast function and bone remodeling. The Journal of biological chemistry 279: 27560–27566. doi:10.1074/jbc.M404222200.

7. Wang H-S, Hung Y, Su C-H, Peng S-T, Guo Y-J, et al. (2005) CD44 cross-linking induces integrin-mediated adhesion and transendothelial migration in breast cancer cell line by up-regulation of LFA-1 (alpha L beta2) and VLA-4 (alpha4beta1). Experimental cell research 304: 116–126. doi:10.1016/j.yexcr.2004.10.015.

8. Eppert K, Wunder JS, Aneliunas V, Kandel R, Andrulis IL (2005) von Willebrand factor expression in osteosarcoma metastasis. Modern Pathology 18: 388–397. doi:10.1038/modpathol.3800265.
